# Association between organophosphorus pesticide exposure and the prevalence of kidney stones in US adults: A population-based study

**DOI:** 10.1097/MD.0000000000043227

**Published:** 2025-07-11

**Authors:** Yao Peng, Shugen Li, Jie Jiang, Xiaoting Lu, Lanxiang Liu, Guangchun Wang, Shang Gao

**Affiliations:** a School of Clinical Medicine, China Medical University, Shenyang, China; b Department of Urology, Suzhou Wuzhong No. 2 People’s Hospital, Suzhou, China; c Department of Urology, Shanghai Tenth People’s Hospital, School of Medicine, Tongji University, Shanghai, China.

**Keywords:** a cross-sectional survey, glyphosate, kidney stones, National Health and Nutrition Examination Survey

## Abstract

It has been demonstrated that exposure to glyphosate (GLY) may result in the development of a number of diseases. The objective of this study was to ascertain whether there is a correlation between GLY exposure and the risk of developing kidney stones in adults residing in the United States. A cross-sectional study was conducted on 4178 patients from the 2013 to 2018 National Health and Nutrition Examination Survey. To explore the association between GLY exposure and the risk of developing kidney stones, dose–response analysis curves with restricted cubic barplots, logistic regression, propensity score matching, and subgroup analyses were employed. Of the 4178 participants, 443 self-reported having kidney stones. After adjusting for sex, age, race, education level, marital status, body mass index, hypertension, diabetes mellitus, recreational activities, and smoking and drinking status, we found that GLY exposure was positively associated with the risk of kidney stone prevalence. The patients were divided into 4 groups based on quartiles of urinary GLY levels and a logistic regression analysis was performed after adjusting for potential confounders. This analysis demonstrated a positive association between GLY exposure and the risk of kidney stones when compared with Q1 (OR = 1). The results indicated that the risk of developing kidney stones increased with increasing urinary GLY concentrations. The present study found a positive association between urinary GLY levels and the risk of developing kidney stones. This association can be prevented by reducing occupational exposure to GLY.

## 1. Introduction

Kidney stones, a major public health concern worldwide, have seen a rise in both prevalence and incidence over the past few decades. In the United States, the occurrence of kidney stones in adults has nearly tripled, increasing from 3.8% in 1976 to 11.0% in 2018.^[1]^ These stones are essentially mineral accumulations or adhesions found in the renal pelvis or calyces.^[2]^ Being a systemic disorder, kidney stones heighten the risk of various conditions including chronic kidney disease,^[3]^ bone loss, fractures,^[4]^ coronary artery disease, hypertension, type 2 diabetes, and metabolic syndrome.^[5]^ Early intervention in the case of kidney stones can alleviate the socio-economic impact they impose.^[6,7]^ Hence, gaining an understanding of the alterable risk factors associated with kidney stones is crucial, enabling healthcare professionals to more effectively assist their patients in preventing and managing this condition.

Glyphosate (GLY), a wide-ranging organophosphorus pesticide,^[8]^ is the primary component in GLY-based herbicides. It can be found in the air, water,^[9]^ food supply,^[10]^ and even in biological fluids like urine,^[11]^ blood,^[12]^ and breast milk.^[13]^ The pervasive use of GLY has led to an increase in residues, posing a substantial public health risk due to exposure via food and water.^[14]^ Research indicates that GLY negatively impacts both cancerous and noncancerous health outcomes, including respiratory ailments, spontaneous miscarriages, and developmental attention deficit disorders.^[15]^ Moreover, animal studies have revealed neurotoxic effects from high-exposure levels to GLY, such as disruption of developmental and neurotransmission processes, heightened oxidative stress, and inflammation, ultimately resulting in neuronal death and behavioral alterations.^[16–18]^ It is worth mentioning that the majority of these investigations have been carried out on animals or populations exposed due to their occupation. As a result, we currently lack data on the impact of GLY exposure on the incidence of kidney stones in the broader population.

The purpose of this study was to explore the impact of varying degrees of GLY exposure on the occurrence of kidney stones within the population. Specifically, the study sought to uncover any potential links between the concentration of GLY in urine and the incidence of kidney stones within a substantial demographic in the United States. To accomplish this, the study utilized the most recent data from the National Health and Nutrition Examination Survey (NHANES) up until January 2024.

## 2. Materials and methods

### 2.1. Data source and study population

NHANES is a biennial, multi-stage survey run by the National Center for Health Statistics under the Centers for Disease Control and Prevention. It assesses the health and nutrition of noninstitutionalized U.S. residents and gathers data on demographics, environmental exposure, and health conditions. The information collected is publicly accessible. For detailed methodology and participant eligibility criteria, please refer to the NHANES official website: https://wwwn.cdc.gov/nchs/nhanes/continuousnhanes/default.aspx.

**Figure 1.**
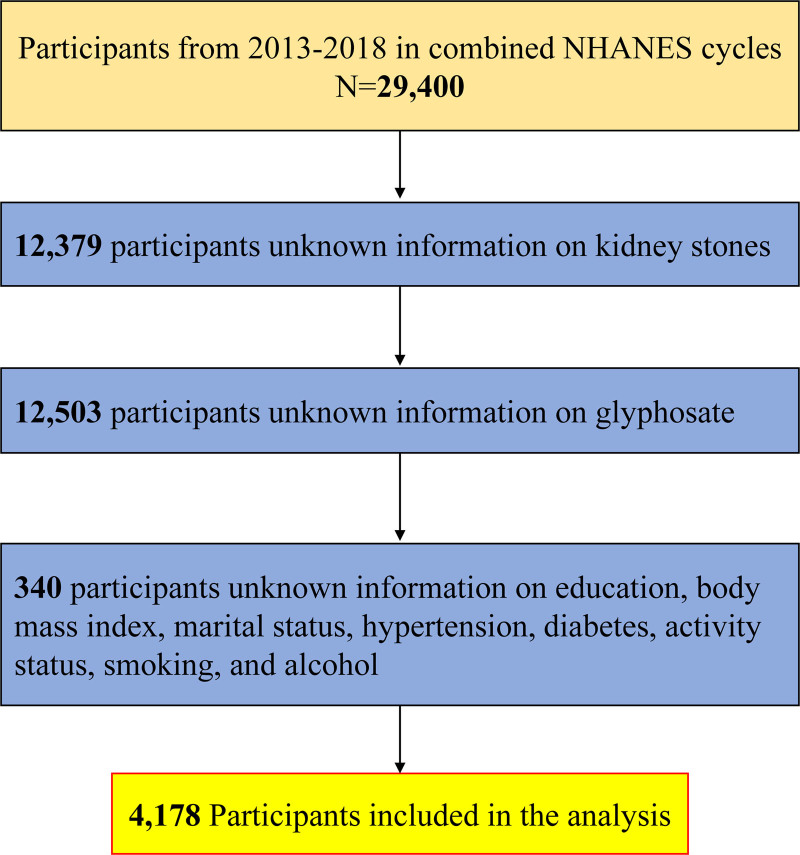
Study population screening flowchart. NHANES = National Health and Nutrition Examination Survey.

The study utilized surveys from 3 NHANES cycles (2013–2014, 2015–2016, and 2017–2018) as they were the only ones with both urine GLY and kidney stone data. The NHANES data collection methodology was approved by the National Center for Health Statistics Research Ethics Review Board under Protocol #2011-17 and Protocol #2018-01 for the years 2013 to 2018. Out of the total sample (n = 29,400), 24,882 participants were excluded due to the absence of kidney stones and urine GLY data in these cycles. We excluded 340 participants who were missing data on education, body mass index, marital status, hypertension, diabetes, activity status, smoking, and alcohol use. The remaining 4178 participants were included for further analysis, as shown in Figure 1.

### 2.2. Measurement of urinary GLY

The NHANES studies conducted between 2013 and 2018 collected random urine samples from participants, including a subset of individuals aged 6 years and older. These samples underwent analysis using 2D-on-line ion chromatography, tandem mass spectrometry, and isotope dilution quantification. The concentration of GLY was measured in these samples and expressed as ng/mL. The lower limit of detection (LLOD) for GLY was set at 0.2 ng/mL. For GLY levels below the LLOD, an imputed value was used, calculated as the LLOD divided by the square root of 2, following NHANES guidelines. The detailed methodology is available on the NHANES website. The studies found that a considerable proportion of the urine samples contained GLY concentrations at or above the detection limit.

### 2.3. Assessment of kidney stones

The presence of kidney stones in participants was determined using the NHANES Kidney Status Questionnaire. Participants who answered “yes” to the question “Have you ever had a kidney stone?” were considered to have a history of kidney stones. Previous studies have confirmed the reliability of this questionnaire.^[19]^

### 2.4. Other covariates

Additionally, we included other confounding variables, such as sex (male, female), age, race (Mexican American, non-Hispanic White, non-Hispanic Black, other Hispanic, and others), educational level (≤high school graduate and >high school graduate), marital status (married and unmarried), body mass index, hypertension (yes, no), diabetes (yes, no), vigorous recreational activity (yes, no), recreational activity (yes, no), smoking (<100 cigarettes in life, ≥100 cigarettes in life), and alcohol (<12 drinks/year, ≥12 drinks/year). Moreover, hypertension and diabetes are defined as conditions diagnosed by physicians, based on their self-reported assessments. Alcohol is defined by this question: “Had at least 12 alcohol drinks/1 year?,” and smoking is defined by this question: “Smoked at least 100 cigarettes in life.” In order to control for the effect of confounders on outcome variables, matching variables for propensity score matching were selected. These included gender, age, race, education level, marital status, personal exercise habits, and underlying disease. These variables were selected based on available evidence and clinical relevance. The matching process was conducted on a 1:1 basis to ensure that all standardized differences in the covariates between the matched groups were <0.1. Following the matching process, the sample size was reduced from 4178 to 3724 with a view to balancing statistical precision and bias control.

### 2.5. Statistical analysis

This study employed the weighted sampling of NHANES to interpret the research design. Continuous variables that adhered to a normal distribution were represented by the mean ± standard deviation. For the analysis of continuous and categorical variables, and to ascertain their statistical differences, a weighted *t* test and a chi-square test were utilized respectively. One-way ANOVA was used to compare the difference in means of the 4 groups. The association between urinary GLY concentration and the prevalence of kidney stones was evaluated using binary logistic regression analysis. In the multifactor logistic regression model and the propensity score weighted model, our base model incorporated gender, age, race, education levels, and marital status for correction. Our core model incorporated base model plus body mass index, vigorous recreational activity, moderate recreational activity, smoking, and alcohol for correction. Our extended model incorporated core model plus hypertension and diabetes were corrected to control for their potential confounding effects on the risk of kidney stone occurrence. A restricted cubic spline function was implemented to depict the dose–response correlation between GLY and kidney stones. The statistical analyses were executed using SPSS (version 24.0) and R (version 4.1.3) software. All graphical representations were generated using R (version 4.1.3) and Adobe Illustrator (version 26.0) software. A *P*-value <.05 (two-tailed) was deemed to indicate statistical significance.

## 3. Results

### 3.1. Participant characteristics

Based on the inclusion and exclusion criteria presented in Figure 1, this study included 4178 participants from the NHANES database between 2013 and 2018. Table 1 displays the clinical baseline characteristics of all patients, with 3735 (89.4%) having no history of kidney stones and 443 (10.6%) having a history of kidney stones. The clinical characteristics of all subjects were assessed using a chi-square test and *t* test. The study found statistically significant differences between nonrenal stone patients and renal stone patients in terms of age (*P* < .001), race (*P* < .001), marital status (*P* = .001), body mass index (*P* < .001), hypertension (*P* < .001), diabetes mellitus (*P* < .001), strenuous recreational activities (*P* < .001), moderate recreational activities (*P* < .001), smoking (*P* = .005), and GLY (*P* < .001). It was found that patients with a history of kidney stones had a higher prevalence of the condition among males, non-Hispanic Whites, those with a history of hypertension, and those who lacked recreational activities. Additionally, smoking and alcohol consumption were also associated with a higher prevalence of kidney stones. The age distribution of these patients was 55 years.

**Table 1:** Baseline characteristics of NHANES participants between 2013 to 2018 (n = 4178).*

Characteristic	Overall	None kidney stone	Kidney stone	t or χ^2^	*P*-value
Sex (N, %)				1.891	.169
Male	2059 (49.3)	1827 (88.7)	232 (11.3)		
Female	2119 (50.7)	1908 (90.0)	211 (10.0)		
Age (mean ± SD)	50.12 ± 17.54	49.46 ± 17.59	55.70 ± 16.06	-7.661	<.001
Race (N, %)				43.205	<.001
Mexican American	616 (14.7)	550 (89.3)	66 (10.7)		
Other Hispanic	427 (10.2)	380 (89.0)	47 (11.0)		
Non-Hispanic White	1687 (40.4)	1453 (86.1)	234 (13.9)		
Non-Hispanic Black	835 (20.0)	780 (93.4)	55 (6.6)		
Other race	613 (14.7)	572 (93.3)	41 (6.7)		
Education level (N, %)				0.001	.982
≤ High school graduate	1794 (42.9)	1604 (89.4)	190 (10.6)		
>High school graduate	2384 (57.1)	2131 (89.4)	253 (10.6)		
Marital status (N, %)				11.670	.001
Married	2160 (51.7)	1897 (87.8)	263 (12.2)		
Unmarried	2018 (48.3)	1838 (91.1)	180 (8.9)		
Body mass index (kg/m^2^) (mean ± SD)	29.64 ± 7.3	29.44 ± 7.30	31.28 ± 7.17	-5.029	<.001
Hypertension (N, %)				42.126	<.001
Yes	1561 (37.4)	1333 (85.4)	228 (14.6)		
No	2617 (62.6)	2402 (91.8)	215 (8.2)		
Diabetes (N, %)				46.097	<.001
Yes	569 (13.6)	464 (81.5)	105 (18.5)		
No	3491 (83.6)	3170 (90.8)	321 (9.2)		
Borderline	118 (2.8)	101 (85.6)	17 (14.4)		
Vigorous recreational activity (N, %)				16.141	<.001
Yes	969 (23.2)	900 (92.9)	69 (7.1)		
No	3209 (76.8)	2835 (88.3)	347 (11.7)		
Moderate recreational activity (N, %)				12.116	<.001
Yes	1756 (42.0)	1604 (91.3)	152 (8.7)		
No	2422 (58.0)	2131 (88.0)	291 (12.0)		
Smoking (N, %)				7.977	.005
< 100 cigarettes in life	2338 (56.0)	2118 (90.6)	220 (9.4)		
≥100 cigarettes in life	1840 (44.0)	1617 (87.9)	223 (12.1)		
Alcohol (N, %)				0.005	.945
< 12 drinks/year	958 (22.9)	857 (89.5)	101 (10.5)		
≥12 drinks/year	3220 (77.1)	2878 (89.4)	342 (10.6)		
Glyphosate (ng/mL) (mean ± SD)	0.49 ± 0.59	0.48 ± 0.58	0.57 ± 0.61	-2.934	<.001

NHANES = National Health and Nutrition Examination Survey, SD = standards deviation.

* For categorical variables, *P* values were analyzed by chi-square tests. For continuous variables, the *t* test for slope was used in generalized linear models. Values are presented as means + SD or %.

We then further analyzed the clinical characteristics of all participants by quartile level of GLY (Q1, <0.141 ng/mL; Q2, 0.141–0.326 ng/mL; Q3, 0.326–0.569 ng/mL; Q4, ≥0.569 ng/mL) (Table 2). The results showed that being male (54.2%), non-Hispanic White (42.9%), >high school graduate education (55.2%), no history of hypertension (59.2%), no history of diabetes mellitus (80.0%), no strenuous recreational activity (78.9%), no moderate recreational activity (60.8%), lifetime smoking < 100 cigarettes (53.9%), and ≥ 12 drinks per year (76.6%) were associated with the highest levels of GLY concentrations.

**Table 2:** Characteristics of the study population by glyphosate in NHANES participants between 2013 and 2018.*

Characteristic	Glyphosate (ng/mL)					
	Q1	Q2	Q3	Q4	F or χ^2^	*P*-value
	N = 1135	N = 952	N = 1045	N = 1046		
Sex (N, %)					21.582	<.001
Male	524 (25.4)	433 (21.0)	535 (26.0)	567 (27.5)		
Female	611 (28.8)	519 (24.5)	510 (24.1)	479 (22.6)		
Age (mean ± SD)	48.46 ± 16.74	49.62 ± 17.17	49.58 ± 17.70	52.91 ± 18.28	12.886	<.001
Race (N, %)					58.342	<.001
Mexican American	177 (28.7)	143 (23.2)	170 (27.6)	126 (20.5)		
Other Hispanic	121 (28.3)	106 (24.8)	93 (21.8)	107 (25.1)		
Non-Hispanic White	408 (24.2)	389 (23.1)	441 (26.1)	449 (26.6)		
Non-Hispanic Black	201 (24.1)	181 (21.7)	217 (26.0)	236 (28.3)		
Other race	228 (37.2)	133 (21.7)	124 (20.2)	128 (20.9)		
Education level (N, %)					5.513	.138
≤High school graduate	456 (25.4)	409 (22.8)	461 (25.7)	468 (26.1)		
>High school graduate	679 (28.5)	543 (22.8)	584 (24.5)	578 (24.2)		
Marital status (N, %)					1.503	.681
Married	595 (27.5)	494 (22.9)	547 (25.3)	524 (24.3)		
Unmarried	504 (26.8)	458 (22.7)	498 (24.7)	522 (25.9)		
Body mass index (kg/m^2^) (mean ± SD)	28.69 ± 6.79	29.89 ± 7.61	29.83 ± 7.19	30.25 ± 7.57	9.494	<.001
Hypertension (N, %)					10.865	.012
Yes	389 (24.9)	345 (22.1)	400 (25.6)	427 (27.4)		
No	746 (28.5)	607 (23.2)	645 (24.6)	619 (23.7)		
Diabetes (N, %)					38.744	<.001
Yes	116 (20.4)	105 (18.5)	158 (27.8)	190 (33.4)		
No	991 (28.4)	815 (23.3)	859 (24.6)	826 (23.7)		
Borderline	28 (23.7)	32 (27.1)	28 (23.7)	30 (25.4)		
Vigorous recreational activity (N, %)					4.800	.187
Yes	284 (29.3)	225 (23.2)	239 (24.7)	221 (22.8)		
No	851 (26.5)	727 (22.7)	806 (25.1)	825 (25.7)		
Moderate recreational activity (N, %)					5.121	.163
Yes	497 (28.3)	407 (23.2)	442 (25.2)	410 (23.3)		
No	638 (26.3)	545 (22.5)	603 (24.9)	636 (26.3)		
Smoking (N, %)					3.848	.278
<100 cigarettes in life	656 (28.1)	541 (23.1)	577 (24.7)	564 (24.1)		
≥100 cigarettes in life	479 (26.0)	411 (22.3)	468 (25.4)	482 (26.2)		
Alcohol (N, %)					10.931	.012
<12 drinks/year	266 (27.8)	183 (19.1)	264 (27.6)	245 (25.6)		
≥12 drinks/year	869 (27.0)	769 (23.9)	781 (24.3)	801 (24.9)		

Q1, < 0.141 ng/mL; Q2, 0.141–0.326 ng/mL; Q3, 0.326–0.569 ng/mL; Q4, ≥ 0.569 ng/mL.

NHANES = National Health and Nutrition Examination Survey, SD = standard deviation.

* For categorical variables, *P* values were analyzed by chi-square tests. For continuous variables, the *t* test for slope was used in generalized linear models. One-way ANOVA was used to compare the difference in means of the 4 groups. Values are presented as means + SD or %.

### 3.2. GLY and kidney stones

**Figure 2.**
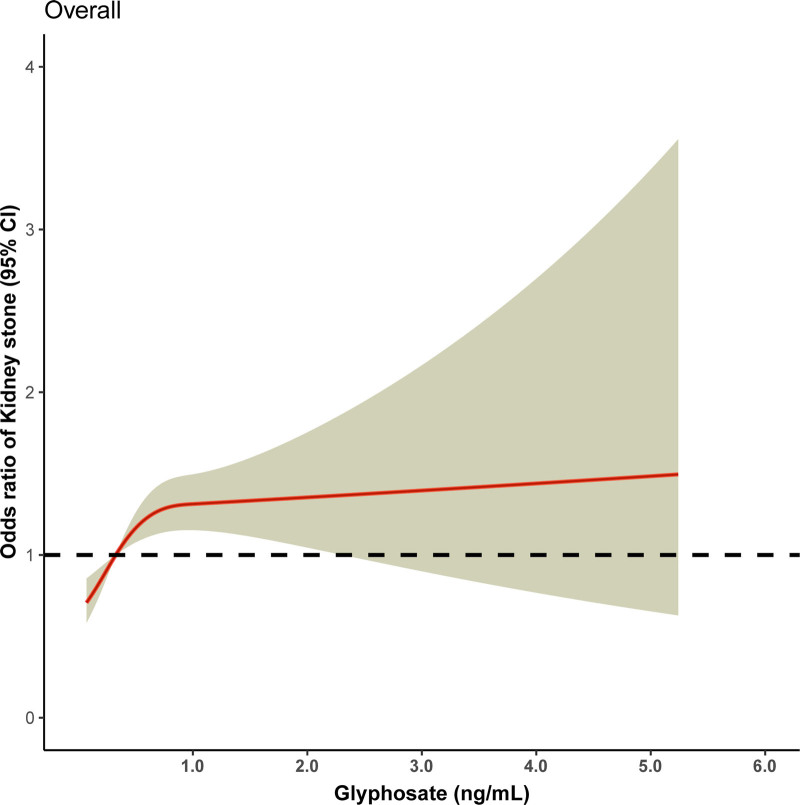
Dose–response curve for the risk of developing kidney stones from glyphosate exposure, with yellow shading representing the 95% CI.

**Figure 3.**
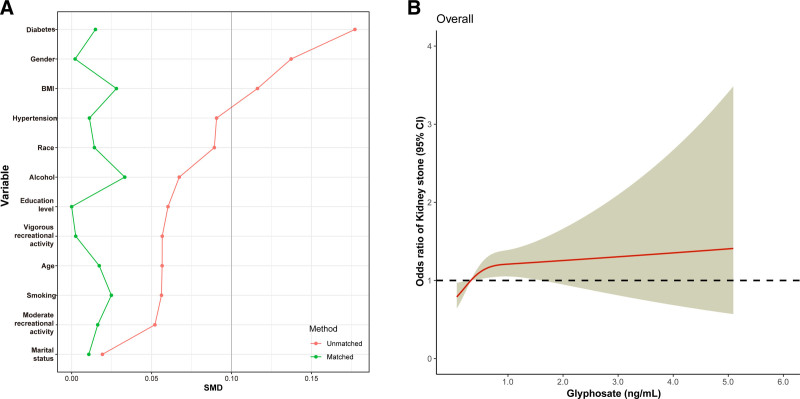
After conducting propensity score matching (PSM) of participants, this study examines the relationship between glyphosate exposure and the risk of developing kidney stones. (A) The participants were matched based on their propensity scores. The red lines indicate the results before the matching, while the green lines indicate the results after the matching. The vertical coordinates represent the distribution of variables. (B) Association between exposure to glyphosate and the risk of developing kidney stones after PSM.

After adjustment for covariates, the odds ratio (95% confidence interval) for kidney stones increased with increasing urinary GLY levels (Fig. 2), reaching a maximum at a urinary GLY concentration of 5.24 ng/mL [1.50 (0.63–3.55) overall (*P*_for nonlinearity_ = .002)]. Logistic regression analysis was used to further identify risk factors associated with the prevalence of kidney stones. After adjusting for sex, age, race, education, marital status, body mass index, hypertension, diabetes mellitus, recreational activities, smoking and drinking status, we found that urinary GLY was significantly and positively associated with the risk of kidney stones. Covariates were adjusted in 3 models: basic model: gender, age, race, education levels, and marital status; core model: basic model plus, body mass index, vigorous recreational activity, moderate recreational activity, smoking and alcohol; extended model: core model plus hypertension and diabetes (Table 3). Because of the differences between the non-stones and stones groups, we matched participants on propensity scores, and the results after propensity score matching are shown in Figure 3A. Figure 3B shows the dose–response relationship between urinary GLY and kidney stones after propensity score matching. There was a nonlinear relationship between urinary GLY concentration and kidney stone prevalence, and the risk of kidney stone prevalence increased progressively with higher urinary GLY concentration.

**Table 3:** Adjusted odds ratios for associations between the glyphosate and kidney stones in NHANES participants between 2013 to 2018.

Characteristic	N	Basic model		Core model		Extended model	
		aOR (95% CI)	*P*	aOR (95% CI)	*P*	aOR (95% CI)	*P*
Glyphosate							
Q1	1135	1.00	.015	1.00	.038	1.00	.032
Q2	952	1.325 (0.980–1.793)	.068	1.284 (0.984–1.739)	.107	1.311 (0.968–1.776)	.080
Q3	1045	1.385 (1.003–1.858)	.030	1.349 (1.005–1.812)	.046	1.359 (1.013–1.824)	.041
Q4	1046	1.598 (1.200–2.128)	.001	1.523 (1.142–2.030)	.004	1.537 (1.153–2.048)	.003

Basic model: gender, age, race, education levels, and marital status; core model: basic model plus, body mass index, vigorous recreational activity, moderate recreational activity, smoking and alcohol; Extended model: core model plus hypertension and diabetes.

Q1, < 0.141 ng/mL; Q2, 0.141–0.326 ng/mL; Q3, 0.326–0.569 ng/mL; Q4, ≥ 0.569 ng/m.

aOR = adjusted odds ratio, CI = confidence interval, NHANES = National Health and Nutrition Examination Survey, Q = quartile.

### 3.3. Subgroup analyses

**Figure 4.**
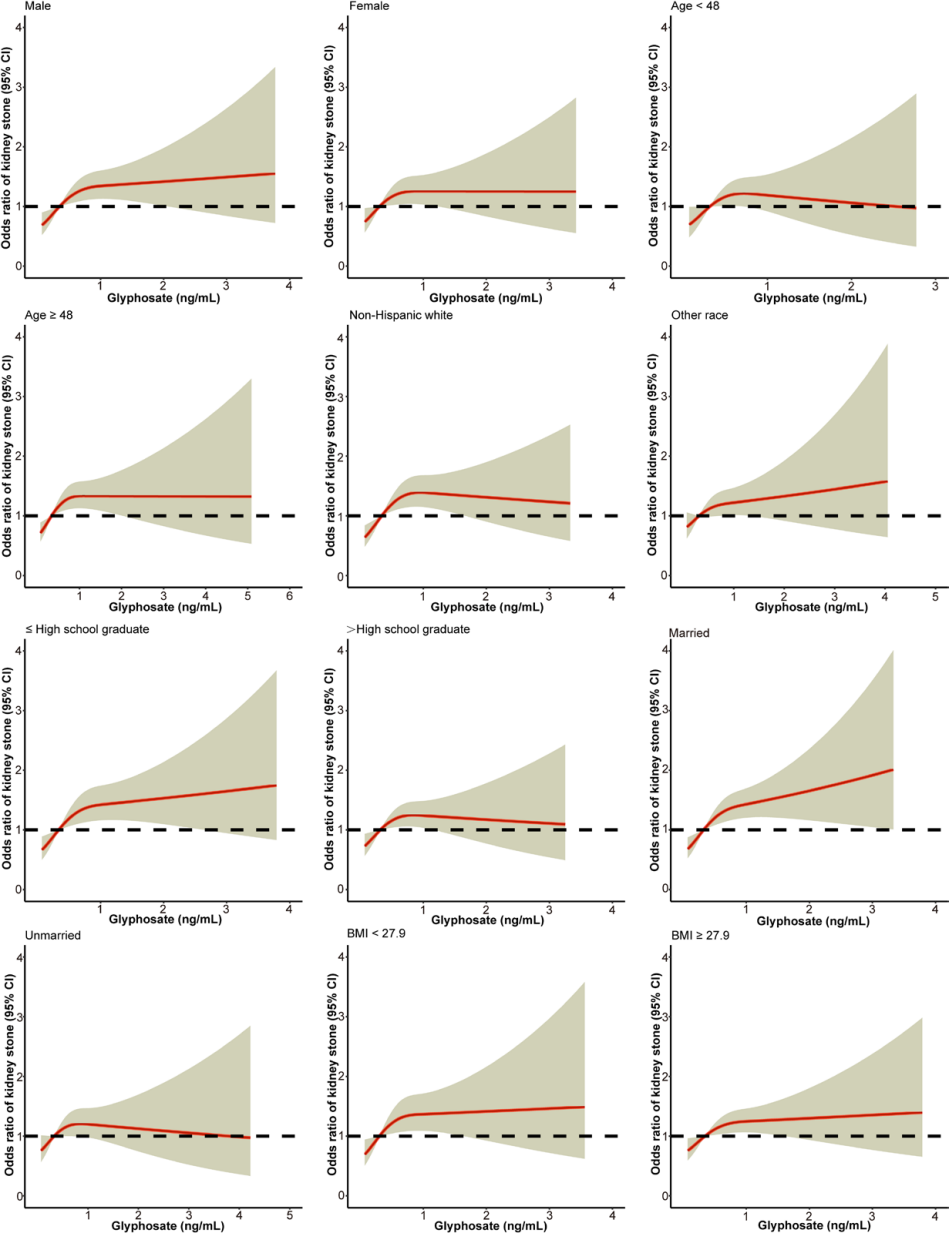
Analysis of the dose–response relationship between glyphosate exposure and the prevalence of kidney stones in subgroups of gender, age, race, educational level, marital status, and BMI; with yellow shading representing the 95% CI. BMI = body mass index, CI = confidence interval.

**Figure 5.**
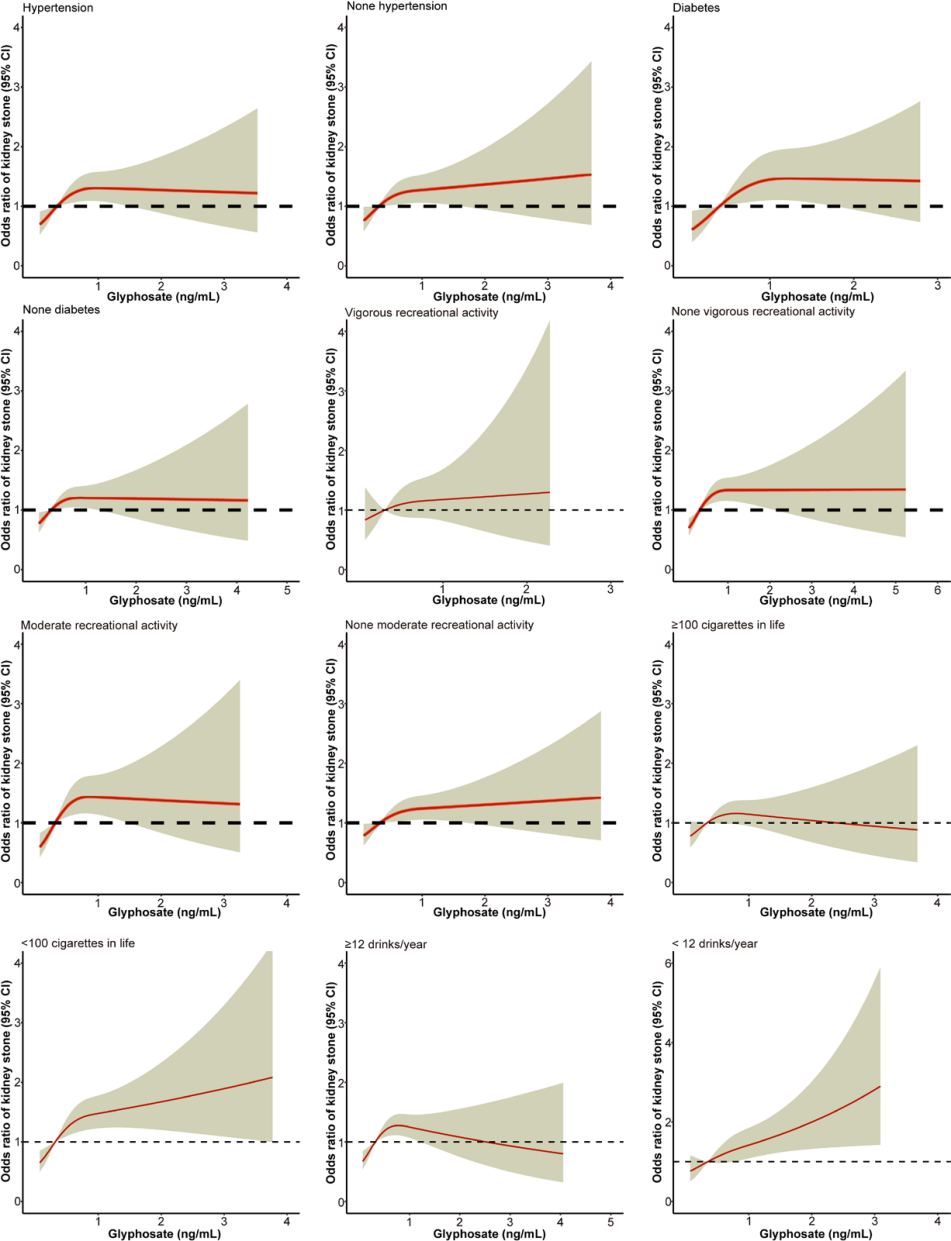
Analysis of the dose–response relationship between glyphosate exposure and the prevalence of kidney stones in subgroups of hypertension, diabetes, recreational activity, smoking, and drinking; with yellow shading representing the 95% CI. CI = confidence interval.

In subgroup analyses (Table 4), urinary GLY levels in Q4 were associated with a higher risk of kidney stones than in Q1 in almost all subgroups, especially among men, non-Hispanic Whites, low educational attainment, married, history of hypertension, history of diabetes mellitus, moderate recreational activity, and one-year alcohol consumption ≥ 12 (all *P* < .05). The dose–response curves from the subgroup analyses also showed that the risk of developing kidney stones was positively correlated with urinary GLY levels (Figs. 4 and 5).

**Table 4:** Subgroup analyses between the glyphosate and kidney stones in NHANES participants between 2013 to 2018.*

Subgroups	Total glyphosate [aOR (95% CI)]			
	Q1	Q2	Q3	Q4
	N = 1135	N = 952	N = 1045	N = 1046
Sex				
Male	1.000	1.518 (0.973–2.366)	1.434 (0.936–2.196)	**1.688 (1.121–2.544**)
Female	1.000	1.199 (0.787–1.825)	1.310 (0.868–1.977)	1.397 (0.923–2.114)
Race				
Non-Hispanic White	1.000	1.214 (0.777–1.898)	1.378 (0.902–2.107)	**1.628 (1.075–2.465**)
Other race	1.000	1.431 (0.942–2.175)	1.221 (0.804–1.854)	1.325 (0.882–1.992)
Education level				
≤High school graduate	1.000	1.039 (0.639–1.688)	1.205 (0.762–1.905)	**1.745 (1.134–2.686**)
>High school graduate	1.000	**1.550 (1.047–2.293**)	**1.506 (1.024–2.216**)	1.356 (0.916–2.006)
Marital status				
Married	1.000	1.217 (0.815–1.817)	1.390 (0.950–2.035)	**1.537 (1.055–2.238**)
Unmarried	1.000	1.498 (0.938–2.393)	1.299 (0.814–2.074)	1.515 (0.964–2.381)
Hypertension				
Yes	1.000	**1.710 (1.097–2.665**)	1.496 (0.967–2.313)	**1.873 (1.229–2.854**)
No	1.000	1.027 (0.673–1.567)	1.241 (0.831–1.853)	1.289 (0.864–1.924)
Diabetes				
Yes	1.000	**2.512 (1.128–5.594**)	**1.205 (0.762–1.905**)	**2.897 (1.398–6.004**)
No/borderline	1.000	1.172 (0.842–1.633)	1.171 (0.845–1.624)	1.292 (0.936–1.782)
Vigorous recreational activity				
Yes	1.000	1.481 (0.736–2.978)	0.906 (0.420–1.953)	1.514 (0.744–3.079)
No	1.000	1.283 (0.915–1.798)	**1.460 (1.059–2.013**)	**1.548 (1.128–2.123**)
Moderate recreational activity				
Yes	1.000	1.539 (0.914–2.592)	1.406 (0.842–2.347)	**1.891 (1.149–3.115**)
No	1.000	1.197 (0.822–1.744)	1.339 (0.933–1.922)	1.382 (0.970–1.969)
Smoking				
<100 cigarettes in life	1.000	1.313 (0.847–2.038)	**1.610 (1.063–2.437**)	**1.660 (1.096–2.514**)
≥100 cigarettes in life	1.000	1.293 (0.847–1.975)	1.124 (0.737–1.714)	1.418 (0.947–2.121)
Alcohol				
<12 drinks/year	1.000	1.084 (0.550–2.136)	1.116 (0.605–2.058)	1.508 (0.833–2.732)
≥12 drinks/year	1.000	1.387 (0.985–1.951)	**1.453 (1.037–2.035**)	**1.532 (1.009–2.134**)

Q1, < 0.141 ng/mL; Q2, 0.141–0.326 ng/mL; Q3, 0.326–0.569 ng/mL; Q4, ≥ 0.569 ng/mL.

aOR = adjusted odds ratio, CI = confidence interval, NHANES = National Health and Nutrition Examination Survey, Q = quartile.

* Adjusted covariates: gender, age, race, education levels, marital status, body mass index, vigorous recreational activity, moderate recreational activity, smoking, alcohol, hypertension and diabetes. Bold values means statistical difference.

## 4. Discussion

This study utilized data from NHANES 2013 to 2018 to ascertain the relationship between urinary GLY concentration and the prevalence of kidney stones in a large population. The findings of this study provide novel epidemiological evidence for the association between environmental pollutants and urological diseases. Following adjustment for demographic characteristics, metabolic factors, and behavioral variables, elevated urinary GLY concentrations were found to be significantly associated with an increased risk of kidney stones, and this association was more pronounced in men, non-Hispanic Whites, individuals with low levels of education, and individuals with comorbid metabolic disorders (e.g., hypertension, diabetes). This finding suggests that GLY may contribute to kidney stone formation through oxidative stress or endocrine-disrupting mechanisms. Moreover, genetic predisposition, social–behavioral differences, and metabolic disorders may synergize with environmental exposures to exacerbate the risk of disease. The concomitant distribution of behavioral factors such as physical inactivity, smoking, and alcohol consumption with high GLY exposure and kidney stone risk further supports the potential role of lifestyle interventions (e.g., increased physical activity, control of tobacco, and alcohol intake) in attenuating the harms of environmental toxins, and provides a dual entry point to public health strategies combining both individual behaviors and group protection.

Despite studies indicating that GLY has minimal toxicity in animals and humans,^[20]^ there is mounting evidence suggesting that GLY may have adverse effects on a number of organs, including the kidney,^[21]^ liver,^[22]^ digestive system,^[23]^ cardiovascular system,^[24]^ respiratory system,^[25]^ and reproductive system.^[26]^ Exposure to GLY has been linked to a number of adverse health outcomes, including depression, neurological disorders,^[27]^ cancer, birth defects, and reproductive problems.^[28]^ In 2015, the International Agency for Research on Cancer classified GLY as a probable carcinogen.^[29]^ Some studies have demonstrated that GLY can induce cytotoxicity in human neuroblastoma cells through the activation of apoptosis, autophagy, and necrosis pathways.^[30]^ This finding has been corroborated by recent animal studies, which demonstrated that GLY significantly disrupted brain structure, increased the number of apoptotic cells, and upregulated the expression of inflammatory markers in Wistar rats.^[31]^ Furthermore, postnatal exposure to GLY has been linked to impaired cognitive function and a reduction in the expression of synapse-associated proteins in rats.^[32]^ In summary, GLY displays cytotoxic and genotoxic effects, increases oxidative stress, interferes with the estrogen pathway, induces inflammation and affects lymphocyte function.^[33]^

A number of studies have demonstrated that exposure to GLY can result in renal impairment or the development of chronic kidney disease in individuals^[34–36]^ without an identifiable cause. Furthermore, the mitochondrial toxicity of GLY can result in acute kidney injury.^[37,38]^ Previous animal experiments have identified that GLY can stimulate calcium inward flow and release from intracellular storage compartments in mouse kidney cells. This is achieved by increasing intracellular calcium concentration in HK-2 cells through the activation of N-methyl-D-aspartic acid receptor, as well as by increasing the expression of calcium-sensitive receptors in hard water.^[39,40]^ The disruption of intracellular calcium homeostasis is responsible for inducing endoplasmic reticulum stress.^[40]^ The aforementioned evidence indicates that GLY exposure results in endoplasmic reticulum stress and mitochondrial dysfunction, which ultimately leads to renal tubular injury.

Furthermore, GLY exposure increases lipid peroxidation by increasing the level of inflammation and promoting oxidative stress through the NF-κB signaling pathway.^[41,42]^ Oxidative damage occurs when reactive oxygen species attack the double bonds of unsaturated fats in cell membranes and produce various lipid peroxidation products. Consequently, the rise in reactive oxygen species results in renal cell injury due to the inhibition of cellular respiration, adenosine triphosphate production, the release of cytochrome c from mitochondrial membranes, lipid peroxidation, and the destabilization of cellular membranes, ultimately leading to necrosis and cell death.^[43]^ The aforementioned studies have demonstrated that GLY exposure results in the damage of renal tubular cells, leading to the disruption of their normal function and the disruption of calcium–salt balance homeostasis. This, in turn, causes the formation of kidney stones.

However, the limitations of the study require careful interpretation of the findings. Firstly, the cross-sectional design, despite controlling for confounders through propensity score matching and multiple modeling adjustments, was unable to clarify the temporal causality of GLY exposure and kidney stones. Residual confounders (e.g., unmeasured variables such as dietary calcium intake, water hardness, and so on) may have affected the reliability of the association. Secondly, the GLY data was based on a single urine sample test, which is difficult to reflect long-term cumulative exposure levels. Furthermore, the data covered only 2013 to 2018, which may underestimate the long-term effect of dynamic changes in environmental exposure on disease risk. Furthermore, the NHANES sample is dominated by the U.S. population, and its conclusions require further validation when extrapolated to regions exhibiting significant disparities in GLY use patterns, demographics, and medical diagnostic criteria. Finally, the reliance on self-reporting of kidney stone history may present a risk of misclassification due to underdiagnosis or recall bias in asymptomatic cases. Future studies should therefore employ prospective cohort designs, combining environmental monitoring data (e.g. regional pesticide use) and biospecimen analyses (e.g. stone constituent assays) to elucidate exposure–disease biomechanisms and to develop tailored protection strategies for high-risk populations. At the same time, future research should also combine occupational exposure assessments with environmental and lifestyle data to clarify risks for specific occupations. Cohort studies in high-exposure occupations are needed to verify that the dose–response relationship observed here extends to the occupational setting.

## 5. Conclusion

The present study provides evidence that supports an independent association between elevated urinary GLY levels and an increased risk of kidney stones in U.S. adults (2013–2018), with heightened susceptibility observed in men, non-Hispanic White individuals, and populations with metabolic disorders. While multivariable adjustments and subgroup analyses serve to strengthen the robustness of these findings, the cross-sectional design, reliance on single-time-point exposure assessment, and potential residual confounding preclude causal interpretations. The necessity for future prospective cohort studies incorporating longitudinal biomonitoring, environmental pesticide-use data, and biological characterization of stone composition is indicated, in order to validate the long-term effects of GLY and clarify underlying mechanisms. Public health initiatives ought to accord priority to the mitigation of GLY exposure in high-risk demographics (through the implementation of occupational safety measures, dietary modifications, and lifestyle interventions) with a view to attenuating the synergistic impacts of environmental toxicants and metabolic comorbidities.

## Author contributions

**Conceptualization:** Yao Peng, Jie Jiang, Guangchun Wang, Shang Gao.

**Data curation:** Yao Peng, Shugen Li, Xiaoting Lu, Lanxiang Liu, Guangchun Wang, Shang Gao.

**Formal analysis:** Yao Peng, Shugen Li, Xiaoting Lu, Lanxiang Liu, Guangchun Wang, Shang Gao.

**Investigation:** Yao Peng, Jie Jiang, Guangchun Wang, Shang Gao.

**Methodology:** Shugen Li, Jie Jiang, Lanxiang Liu, Shang Gao.

**Project administration:** Yao Peng, Guangchun Wang, Shang Gao.

**Resources:** Yao Peng, Shugen Li, Jie Jiang, Xiaoting Lu.

**Software:** Yao Peng, Lanxiang Liu, Guangchun Wang.

**Supervision:** Shang Gao.

**Validation:** Yao Peng, Jie Jiang, Xiaoting Lu, Guangchun Wang, Shang Gao.

**Visualization:** Shugen Li, Jie Jiang, Xiaoting Lu, Lanxiang Liu, Guangchun Wang.

**Writing – original draft:** Yao Peng, Shugen Li, Jie Jiang, Xiaoting Lu, Lanxiang Liu, Guangchun Wang, Shang Gao.

**Writing – review & editing:** Yao Peng, Shugen Li, Jie Jiang, Xiaoting Lu, Lanxiang Liu, Guangchun Wang, Shang Gao.

## References

[1] Hill AJ Basourakos SP Lewicki P. Incidence of kidney stones in the United States: the continuous national health and nutrition examination survey. J Urol 2022;207:851–6. 34854755 10.1097/JU.0000000000002331

[2] Khan SR Pearle MS Robertson WG. Kidney stones. Nat Rev Dis Primers 2016;2:16008. 27188687 10.1038/nrdp.2016.8 PMC5685519

[3] Gambaro G Croppi E Bushinsky D. The risk of chronic kidney disease associated with urolithiasis and its urological treatments: a review. J Urol 2017;198:268–73. 28286070 10.1016/j.juro.2016.12.135

[4] Taylor EN Feskanich D Paik JM Curhan GC. Nephrolithiasis and risk of incident bone fracture. J Urol 2016;195:1482–6. 26707509 10.1016/j.juro.2015.12.069 PMC4870104

[5] Sakhaee K Maalouf NM Sinnott B. Clinical review. Kidney stones 2012: pathogenesis, diagnosis, and management. J Clin Endocrinol Metab 2012;97:1847–60. 22466339 10.1210/jc.2011-3492 PMC3387413

[6] Geraghty RM Cook P Walker V Somani BK. Evaluation of the economic burden of kidney stone disease in the UK: a retrospective cohort study with a mean follow-up of 19 years. BJU Int 2020;125:586–94. 31916369 10.1111/bju.14991

[7] Lotan Y Jimenez IB Lenoir-Wijnkoop I. Primary prevention of nephrolithiasis is cost-effective for a national healthcare system. BJU Int 2012;110(11 Pt C):E1060–7. 22686216 10.1111/j.1464-410X.2012.11212.x

[8] Benbrook CM. Trends in glyphosate herbicide use in the United States and globally. Environ Sci Eur 2016;28:3. 27752438 10.1186/s12302-016-0070-0 PMC5044953

[9] Lutri VF Matteoda E Blarasin M. Hydrogeological features affecting spatial distribution of glyphosate and AMPA in groundwater and surface water in an agroecosystem. Cordoba, Argentina. Sci Total Environ 2020;711:134557. 31812431 10.1016/j.scitotenv.2019.134557

[10] Bohn T Cuhra M Traavik T Sanden M Fagan J Primicerio R. Compositional differences in soybeans on the market: glyphosate accumulates in Roundup Ready GM soybeans. Food Chem 2014;153:207–15. 24491722 10.1016/j.foodchem.2013.12.054

[11] Gillezeau C van Gerwen M Shaffer RM. The evidence of human exposure to glyphosate: a review. Environ Health 2019;18:2. 30612564 10.1186/s12940-018-0435-5 PMC6322310

[12] Kongtip P Nankongnab N Phupancharoensuk R. Glyphosate and paraquat in maternal and fetal serums in Thai women. J Agromedicine 2017;22:282–9. 28422580 10.1080/1059924X.2017.1319315

[13] Camiccia M Candiotto L Gaboardi SC Panis C Kottiwitz L. Determination of glyphosate in breast milk of lactating women in a rural area from Parana state, Brazil. Braz J Med Biol Res 2022;55:e12194. 35894382 10.1590/1414-431X2022e12194 PMC9322831

[14] Van Bruggen A He MM Shin K. Environmental and health effects of the herbicide glyphosate. Sci Total Environ 2018;616-617:255–68. 29117584 10.1016/j.scitotenv.2017.10.309

[15] Mink PJ Mandel JS Lundin JI Sceurman BK. Epidemiologic studies of glyphosate and non-cancer health outcomes: a review. Regul Toxicol Pharmacol 2011;61:172–84. 21798302 10.1016/j.yrtph.2011.07.006

[16] Bali YA Ba-Mhamed S Bennis M. Behavioral and immunohistochemical study of the effects of subchronic and chronic exposure to glyphosate in mice. Front Behav Neurosci 2017;11:146. 28848410 10.3389/fnbeh.2017.00146 PMC5550406

[17] Ait-Bali Y Ba-M’Hamed S Gambarotta G Sassoe-Pognetto M Giustetto M Bennis M. Pre- and postnatal exposure to glyphosate-based herbicide causes behavioral and cognitive impairments in adult mice: evidence of cortical ad hippocampal dysfunction. Arch Toxicol 2020;94:1703–23. 32067069 10.1007/s00204-020-02677-7

[18] Costas-Ferreira C Duran R Faro L. Toxic effects of glyphosate on the nervous system: a systematic review. Int J Mol Sci 2022;23:4605. 35562999 10.3390/ijms23094605 PMC9101768

[19] Taylor EN Stampfer MJ Curhan GC. Obesity, weight gain, and the risk of kidney stones. JAMA 2005;293:455–62. 15671430 10.1001/jama.293.4.455

[20] Williams GM Kroes R Munro IC. Safety evaluation and risk assessment of the herbicide Roundup and its active ingredient, glyphosate, for humans. Regul Toxicol Pharmacol 2000;31(2 Pt 1):117–65. 10854122 10.1006/rtph.1999.1371

[21] Babich R Ulrich JC Ekanayake EMDV. Kidney developmental effects of metal-herbicide mixtures: Implications for chronic kidney disease of unknown etiology. Environ Int 2020;144:106019. 32818823 10.1016/j.envint.2020.106019

[22] Endirlik BU Bakir E Okcesiz A. Investigation of the toxicity of a glyphosate-based herbicide in a human liver cell line: assessing the involvement of Nrf2 pathway and protective effects of vitamin E and alpha-lipoic acid. Environ Toxicol Pharmacol 2022;96:103999. 36252731 10.1016/j.etap.2022.103999

[23] de Maria Serra F Parizi JLS de Mello Odorizzi GAS. Subchronic exposure to a glyphosate-based herbicide causes dysplasia in the digestive tract of Wistar rats. Environ Sci Pollut Res Int 2021;28:61477–96. 34173954 10.1007/s11356-021-15051-6

[24] Maia FCC Porto RA Magalhaes LR Chagas PHN Nai GA. Cardiovascular damage associated with subchronic exposure to the glyphosate herbicide in Wistar rats. Toxicol Ind Health 2021;37:210–8. 33625310 10.1177/0748233721996578

[25] Pandher U Kirychuk S Schneberger D. Pulmonary inflammatory response from co-exposure to LPS and glyphosate. Environ Toxicol Pharmacol 2021;86:103651. 33812014 10.1016/j.etap.2021.103651

[26] Liu JB Li ZF Lu L Wang ZY Wang L. Glyphosate damages blood-testis barrier via NOX1-triggered oxidative stress in rats: long-term exposure as a potential risk for male reproductive health. Environ Int 2022;159:107038. 34906888 10.1016/j.envint.2021.107038

[27] Chang MH Chu PL Wang C Lin CY. Association between glyphosate exposure and erythrograms in a representative sample of US adults: NHANES 2013-2014. Environ Sci Pollut Res Int 2023;30:91207–15. 37474857 10.1007/s11356-023-28905-y

[28] de Araujo JS Delgado IF Paumgartten FJ. Glyphosate and adverse pregnancy outcomes, a systematic review of observational studies. BMC Public Health 2016;16:472. 27267204 10.1186/s12889-016-3153-3 PMC4895883

[29] Davoren MJ Schiestl RH. Glyphosate-based herbicides and cancer risk: a post-IARC decision review of potential mechanisms, policy and avenues of research. Carcinogenesis 2018;39:1207–15. 30060078 10.1093/carcin/bgy105 PMC7530464

[30] Martinez MA Rodriguez JL Lopez-Torres B. Use of human neuroblastoma SH-SY5Y cells to evaluate glyphosate-induced effects on oxidative stress, neuronal development and cell death signaling pathways. Environ Int 2020;135:105414. 31874349 10.1016/j.envint.2019.105414

[31] Adewale OO Adebisi OA Ojurongbe TA Adekomi DA Babatunde IO Adebayo EO. Xylopia aethiopica suppresses markers of oxidative stress, inflammation, and cell death in the brain of Wistar rats exposed to glyphosate. Environ Sci Pollut Res Int 2023;30:60946–57. 37042920 10.1007/s11356-023-26470-y

[32] Luna S Neila LP Vena R Borgatello C Rosso SB. Glyphosate exposure induces synaptic impairment in hippocampal neurons and cognitive deficits in developing rats. Arch Toxicol 2021;95:2137–50. 33837468 10.1007/s00204-021-03046-8

[33] Peillex C Pelletier M. The impact and toxicity of glyphosate and glyphosate-based herbicides on health and immunity. J Immunotoxicol 2020;17:163–74. 32897110 10.1080/1547691X.2020.1804492

[34] Gunarathna S Gunawardana B Jayaweera M Manatunge J Zoysa K. Glyphosate and AMPA of agricultural soil, surface water, groundwater and sediments in areas prevalent with chronic kidney disease of unknown etiology, Sri Lanka. J Environ Sci Health B 2018;53:729–37. 29883246 10.1080/03601234.2018.1480157

[35] Jayasumana C Gunatilake S Siribaddana S. Simultaneous exposure to multiple heavy metals and glyphosate may contribute to Sri Lankan agricultural nephropathy. BMC Nephrol 2015;16:103. 26162605 10.1186/s12882-015-0109-2 PMC4499177

[36] Jayasumana C Paranagama P Agampodi S Wijewardane C Gunatilake S Siribaddana S. Drinking well water and occupational exposure to Herbicides is associated with chronic kidney disease, in Padavi-Sripura, Sri Lanka. Environ Health 2015;14:6. 25596925 10.1186/1476-069X-14-6 PMC4417209

[37] Kronberg MF Clavijo A Moya A. Glyphosate-based herbicides modulate oxidative stress response in the nematode *Caenorhabditis elegans*. Comp Biochem Physiol C Toxicol Pharmacol 2018;214:1–8. 30142450 10.1016/j.cbpc.2018.08.002

[38] Luo L Wang F Zhang Y Zeng M Zhong C Xiao F. In vitro cytotoxicity assessment of roundup (glyphosate) in L-02 hepatocytes. J Environ Sci Health B 2017;52:410–7. 28281894 10.1080/03601234.2017.1293449

[39] Gao H Chen J Ding F. Activation of the N-methyl-d-aspartate receptor is involved in glyphosate-induced renal proximal tubule cell apoptosis. J Appl Toxicol 2019;39:1096–107. 30907447 10.1002/jat.3795

[40] Wang R Chen J Ding F. Renal tubular injury induced by glyphosate combined with hard water: the role of cytosolic phospholipase A2. Ann Transl Med 2021;9:130. 33569432 10.21037/atm-20-7739 PMC7867956

[41] Prasad M Gatasheh MK Alshuniaber MA. Impact of glyphosate on the development of insulin resistance in experimental diabetic rats: role of NFkappaB signalling pathways. Antioxidants (Basel) 2022;11:2436. 36552644 10.3390/antiox11122436 PMC9774325

[42] Sidthilaw S Sapbamrer R Pothirat C Wunnapuk K Khacha-Ananda S. Effects of exposure to glyphosate on oxidative stress, inflammation, and lung function in maize farmers, Northern Thailand. BMC Public Health 2022;22:1343. 35836163 10.1186/s12889-022-13696-7 PMC9281059

[43] Bhatia D Capili A Choi ME. Mitochondrial dysfunction in kidney injury, inflammation, and disease: potential therapeutic approaches. Kidney Res Clin Pract 2020;39:244–58. 32868492 10.23876/j.krcp.20.082 PMC7530368

